# Significant survival improvement of patients with recurrent breast cancer in the periods 2001-2008 vs. 1992-2000

**DOI:** 10.1186/1471-2407-11-118

**Published:** 2011-03-31

**Authors:** Hideo Shigematsu, Hidetoshi Kawaguchi, Yoshiaki Nakamura, Kimihiro Tanaka, Satoko Shiotani, Chinami Koga, Sumiko Nishimura, Kenichi Taguchi, Kenichi Nishiyama, Shinji Ohno

**Affiliations:** 1 Department of Breast Surgery, Hiroshima University Hospital, Hiroshima, Japan; 2 Department of Breast Oncology, National Kyushu Cancer Center, Fukuoka, Japan; 3 Department of Pathology, National Kyushu Cancer Center, Fukuoka, Japan

## Abstract

**Background:**

It is unclear whether individualized treatments based on biological factors have improved the prognosis of recurrent breast cancer. The purpose of this study is to evaluate the survival improvement of patients with recurrent breast cancer after the introduction of third generation aromatase inhibitors (AIs) and trastuzumab.

**Methods:**

A total of 407 patients who received first diagnosis of recurrent breast cancer and treatment at National Kyushu Cancer Center between 1992 and 2008 were retrospectively evaluated. As AIs and trastuzumab were approved for clinical use in Japan in 2001, the patients were divided into two time cohorts depending on whether the cancer recurred before or after 2001. Cohort A: 170 patients who were diagnosed between 1992 and 2000. Cohort B: 237 patients who were diagnosed between 2001 and 2008. Tumor characteristics, treatments, and outcome were compared.

**Results:**

Fourteen percent of cohort A and 76% of cohort B received AIs and/or trastuzumab (P < 0.001). The median overall survival (OS) times after breast cancer recurrence were 1.7 years and 4.2 years for these respective cohorts (P < 0.001). Both the time period and treatment of AIs and/or trastuzumab for recurrent disease were significant prognostic factors in multivariate analysis (cohort B vs. cohort A: HR = 0.70, P = 0.01; AIs and/or trastuzumab for recurrent disease: yes vs. no: HR = 0.46, P < 0.001). When patients were categorized into 4 subgroups by the expression of hormone receptor (HR) and HER-2 status, the median OS times of the HR-positive/HER-2-negative, HR-positive/HER-2-positive, HR-negative/HER-2-positive, and HR-negative/HER-2-negative subtypes were 2.2, 2.4, 1.6, and 1.0 years in cohort A and 4.5, 5.1, 5.0, and 1.4 years in cohort B.

**Conclusions:**

The prognosis of patients with recurrent breast cancer was improved over time following the introduction of AIs and trastuzumab and the survival improvement was apparent in HR- and/or HER-2-positive tumors.

## Background

Molecular targeting therapies have recently become available, and tailored treatments based on individual biological factors have already come to play an important role in breast cancer treatment. In the adjuvant setting, a meta-analysis has shown that 5-year adjuvant treatment with tamoxifen (TAM) reduced the annual risk of recurrence and death by more than 30% in patients with estrogen receptor (ER)-positive tumors [1]. In addition, large randomized controlled trials have shown that third-generation aromatase inhibitors (AIs) are more effective than TAM in post-menopausal early breast cancer patients with HR-positive tumors [2-4]. Among women with HER-2-positive early breast cancer, concurrent or sequential use of trastuzumab with, or after, adjuvant chemotherapy significantly improves both disease-free survival and overall survival rates [5-7]. Adjuvant trastuzumab therapy is expected to decrease the breast cancer mortality rate and, as mentioned above, tailored treatments based on individual biological factors have significantly contributed to the prognostic improvement of patients with early stage breast cancer [8].

Compared with the adjuvant setting, the type of tailored treatments (based on biological factors) that have contributed to the improvement in prognosis for patients with recurrent or advanced breast cancer is less clear. Some retrospective studies have reported that the survival of patients with recurrent breast cancer has improved, over time, with the introduction of new drugs [9-12]. And while it is difficult to ascertain exactly which therapies have contributed to the improved survival of patients with advanced breast cancer [13], the improvement does seem to be associated with the expression of certain biological factors. Andre *et al*. (2004) compared the prognosis of metastatic breast cancer patients over two time periods, and showed a significant prolongation of survival over time in patients with HR-positive tumors [14]. This finding suggests that the improvement was related to therapy targeted at patients who had HR-positive tumors. A recent study of an institutional-based review showed that women with HER2/neu-positive disease who received trastuzumab had improved prognosis compared with women with HER2/neu-negative disease [15]. With the introduction of trastuzumab in daily practice, the survival of patients with HER-2-positive disease may be prolonged overtime. Here, we investigate whether the survival of women with recurrent breast cancer has improved following the introduction of new agents, such as AIs and trastuzumab. The use of these drugs for the treatment of recurrent, or metastatic, breast cancer in Japan was approved in 2001. Thus, we compared the prognosis between patients first diagnosed with recurrent breast before 2001 and those first diagnosed after 2001.

Recent studies have shown that intrinsic subtypes are important prognostic and predictive factors in breast cancer. Thus, in both early and advanced stage breast cancer, the intrinsic subtype has been strongly correlated with prognosis [16-18]. In a neoadjuvant setting, chemosensitivity has been shown to differ among breast cancer subtypes [19,20]. Thus, we also performed an exploratory analysis to determine whether the recent survival improvement in recurrent breast cancer was related to the breast cancer subtype. We classified the patients into four subgroups for this purpose: HR-positive/HER-2-negative; HR-positive/HER-2-positive; HR-negative/HER-2-positive; and HR-negative/HER-2-negative cases. Within each subgroup, we compared the prognosis over time, and evaluated the relationship between the survival improvement and expression of HR and HER-2.

## Methods

All patient data were collected at the Department of Breast Oncology at the National Kyushu Cancer Center, Fukuoka, Japan. This retrospective analysis was performed in accordance with the ethical regulations of the National Kyushu Cancer Center.

### Study Design

A total of 569 patients who were diagnosed and treated for recurrent breast cancer at the National Kyushu Cancer Center between 1992 and 2008 were eligible for this study. All patients had undergone primary surgery for breast cancer without any evidence of distant metastasis and then were clinically determined to have recurrent breast cancer. The diagnosis of recurrent disease was essentially confirmed by physical examination, X-rays, computer tomography (CT), magnetic resonance imaging (MRI), bone scintigraphy and/or other imaging modalities. A biopsy of metastasis was not essential for the diagnosis of recurrent disease. We excluded 133 (23.3%) cases that lacked either HR or HER-2 expression data, and 29 (5.1%) cases that were not followed-up, or who did not receive treatment after first diagnosis of recurrence. The remaining 407 patients were included in the study. Isolated ipsilateral breast cancer recurrence was excluded from the study because it was difficult to distinguish true recurrence from a new primary lesion. The clinicopathological factors and treatments for primary breast cancer and recurrent disease pertaining to these patients were entered prospectively into the hospital database. Patients were assigned to two cohorts based on the time of their first diagnosis of recurrence. Cohort A included patients diagnosed between 1992 and 2000; Cohort B included patients diagnosed between 2001 and 2008. As outlined above, patients were also assigned to four subgroups according to HR and HER-2 expression. The prognosis of the patients within each group was compared between the two time periods. Survival time was defined as the time from the date of first recurrence to the last follow-up examination, or death. Follow-up was ended in December 2009. The primary aims of this study were to evaluate the association between period of diagnosis of recurrent disease and overall survival (OS). As an exploratory analysis, we also determined the relationship between the survival improvement over time and the breast cancer subtype as defined by HR and HER-2 status.

### Variables

Because prognostic variables were unevenly divided between the two main cohorts, a multivariate model was created to determine any association between these cohorts and survival rate, after accounting for other prognostic factors. Information regarding the year of first recurrent diagnosis, patient age, primary tumor size, number of positive axillary lymph nodes, HR status, HER-2 status, relapse free interval (RFI), sites of metastases, brain metastasis, adjuvant therapy, and medical treatments for recurrent disease was obtained directly from the database.

ER and/or progesterone receptor assays were performed using either an enzyme immunoassay method (ELISA) or immunohistochemical analysis (IHC). HER-2 status was estimated by either IHC or fluorescence *in situ *hybridization (FISH) at diagnosis, however most cases were evaluated by IHC. HER-2 FISH were performed for patients in Cohort B. Cases showing overexpression of HER-2/c-erbB2, or HER-2 amplification, were considered to be HER-2-positive. Patients presenting with lung and/or liver and/or brain involvement were classified as having visceral metastases. Patients presenting with other involvements were classified as having non-visceral metastases. There was no limitation on the number or the type of treatments that each patient received.

### Statistical Analysis

Associations between the two cohorts and clinicopathological parameters were analyzed using a Chi-square test for categorical variables, and the Wilcoxon rank sum test for continuous variables. Kaplan-Meier survival curves were plotted, and compared using the log-rank test. Cox proportional hazard models were used for both univariate and multivariate analyses. We analyzed new systemic treatment (AI and/or trastuzumab) for recurrent disease as a confounder in the Cox regression model in order to clarify whether introduction of these new agents was the main contributor to the survival improvement over time. The hazard ratios (95% CI) and P values were reported. All tests were two-sided, and P < 0.05 was considered statistically significant. Statistical analyses were carried out using SPSS II software (Version 11 for Windows; SAS Institute, Tokyo).

## Results

### Patient characteristics

Cohort A contained 170 patients and cohort B contained 237 patients. The median observation time was 2.7 years for cohort A and 3.1 years for cohort B. There have been 286 deaths among the 407 patients. The distribution of patient characteristics within cohorts A and B is shown in Table 1. In all patients, HER-2 status was assessed by IHC and 102 patients (25%) were diagnosed with HER-2/c-erb-B2 overexpressing tumors. Two patients were diagnosed as having HER-2-amplified tumors by fluorescence *in situ *hybridization. The two cohorts were similar in terms of age at diagnosis of recurrence, primary tumor size, HER-2 over-expression, adjuvant treatments, and the site of first recurrence. There were differences in the number of axillary lymph nodes involved (≤ or >3 nodes), HR status, and RFI between the two cohorts. Patients with recurrent disease in cohort B tended to have less lymph node metastases, were more likely to have HR-positive disease, and had a longer disease-free interval compared with those in cohort A. In this study, 3 of the 60 patients with HER-2-positive disease (5%) received trastuzumab and 22 of the 167 patients with HR-positive disease (13%) received aromatase inhibitors as adjuvant therapy in cohort B. In these populations, the effect of these new agents may be attenuated. However, it has been shown that the use of trastuzumab is still effective when continued beyond disease progression [21] and that a change to a different AI is effective after the failure of another AI [22]; therefore, we decided to include these patients in this analysis.

**Table 1:** Patient Characteristics

	Cohort A (n = 170)		Cohort B (n = 237)		Total (n = 407)	
**Characteristic**	**No. of Patients**	**%**	**No. of Patients**	**%**	**No. of Patients**	**%**

Median age, years	51.0		53.8		52.6	

T stage						

T1-T2	126	74.1	178	75.1	304	74.7

T3-T4	40	23.5	51	21.5	91	22.4

Unknown	4	2.4	8	3.4	12	2.9

No. of axillary nodes involved						

≤3 nodes	96	56.5	139	58.6	235	57.7

>3 nodes	70	41.2	80	33.8	150	36.9

Unknown	4	2.4	8	3.4	12	2.9

Hormonal status						

Negative	75	44.1	70	29.5	145	35.6

Positive	95	55.9	167*	70.5	262	64.4

HER-2 status						

Negative	126	74.1	177	74.7	303	74.4

Positive	44	25.9	60	25.3	104	25.6

Subtype						

HR + and HER-2 -	72	42.4	133*	56.1	205	50.4

HR + and HER-2 +	23	13.5	34	14.3	57	14.0

HR - and HER-2 +	21	12.4	26	11.0	47	11.5

HR - and HER-2 -	54	31.8	44*	18.6	98	24.1

Relapse Free Interval						

≤ 2 yr	81	47.6	84*	35.4	165	40.5

>2 yr	89	52.4	153	64.6	242	59.5

Site of first recurrence						

non visceral	77	45.3	124	52.3	201	49.4

visceral	93	54.7	113	47.7	206	50.6

Brain metastasis at diagnosis						

no	163	95.9	224	94.5	387	95.1

yes	6	3.5	12	13.0	18	4.4

Adjuvant chemotherapy						

no	59	34.7	65	27.4	124	30.5

yes	111	65.3	172	72.6	283	69.5

Adjunvant endocrine therapy						

no	80	47.1	92	38.8	172	42.3

yes	90	52.9	145	61.2	235	57.7

*P < .05

Abbreviations: HR, hormone receptor; HER-2, human epidermal growth factor receptor 2

### Treatments received by patients within the two cohorts

The chemotherapy drugs, endocrine therapy agents, and trastuzumab prescribed for the treatment of recurrent disease within the two cohorts are shown in Table 2. Only the agents that were used commonly and approved in Japan were reviewed. Because AI, trastuzumab, capecitabine and vinorelbine were approved after 2001, and taxanes were approved in the late 1990s, there are significant differences in the frequency of use of these agents between the two cohorts. Twenty-two percent of cohort B, and 2.9% of cohort A, were treated with trastuzumab (P < 0.01). In this analysis, most patients with HER-2-positive tumors received trastuzumab-based treatment beyond the time of disease progression and the median number of trastuzumab treatment was 2.1 (range, 1-5). Fifty-six patients with HER-2-positive tumors received trastuzumab treatment. AIs were given to 55% of cohort B and 11% of cohort A (P < 0.01). One hundred forty-four patients with HR-positive tumors received AI treatment. The taxanes (46% vs. 25%, P < 0.01), capecitabine (42% vs. 7.6%, P < 0.01), and vinorelbine (25% vs. 2.4%, P < 0.01) were used more commonly in cohort B. Another trend was the reduced use of anthracyclines (22% vs. 48%, P < 0.01), tamoxifen (26% vs. 38%, P = 0.01), medroxyprogesterone acetate (15% vs. 47%, P < 0.01) and mitomycin (0.8% vs. 49%, P < 0.01) in cohort B.

**Table 2:** Chemotherapies and endocrine therapies for the treatment of recurrent breast cancer for the two cohorts

	Cohort A (n = 170)		Cohort B (n = 237)	
**Therapy**	**No. of Patients**	**%**	**No. of Patients**	**%**

Hormone therapy, %				

Tamoxifen	65	38*	62	26

Medroxyprogesterone acetate	80	47*	35	15

Aromatase inhibitors	19	11*	130	55

Chemotherapy, %				

Anthracyclines	81	48*	51	22

Taxanes	43	25*	110	46

Vinorelbine	4	2.4*	60	25

Capecitabine	13	7.6*	100	42

Mitomycin	82	49*	2	0.8

HER2 targeting therapy, %				

Trastuzumab	5	2.9*	51	22


*P < .05

### Survival of recurrent breast cancer patients within the two cohorts

**Figure 1:**
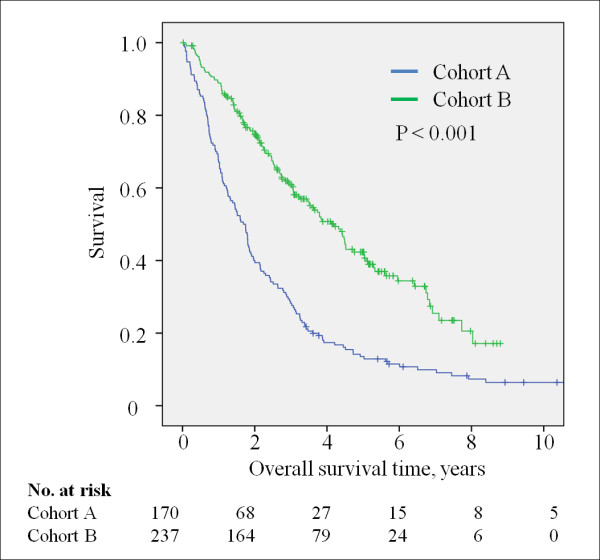
**Kaplan-Meier curves showing the overall survival rates for the two cohorts from the date of diagnosis of recurrent breast cancer**.

In this unadjusted analysis, there was a significant improvement in survival rate for women diagnosed with recurrent breast cancer after 2001. The median survival time increased from 1.7 years in cohort A to 4.2 years in cohort B (Figure 1; P < 0.001). The 3-year survival rate increased from 28% in cohort A to 61% in cohort B, and the 5-year survival rate increased from 12% in cohort A to 41% in cohort B.

Univariate analysis showed that the site of first recurrence, brain metastasis, relapse free interval, hormone status, time period of recurrent breast cancer (pre- or post-2001), T stage, number of involved lymph nodes, adjuvant endocrine therapy, and used of AI and/or trastuzumab for treatment of recurrent disease were all associated with overall survival rates after breast cancer recurrence (Table 3). Because the distribution of patient characteristics differed between the two cohorts, a multivariate analysis was also performed. Both the time period and AI/trastuzumab treatment remained significant prognostic factors (cohort B vs. cohort A: HR = 0.70, P = 0.01; AIs and/or trastuzumab for recurrent disease: yes vs. no: HR = 0.46, P < 0.001). The presence of visceral metastases, brain metastasis, HR status, and T stage and the number of axillary metastases were also found to be significant adverse prognostic factors. HER-2 status was associated with prognosis in the multivariate analysis and adjuvant endocrine therapy was not associated with survival after disease recurrence.

**Table 3:** Cox univariate and multivariate analysis of the survival in recurrent breast cancer

Characteristics	Univariate analysis			Multivariate analysis		
	**HR**	**95% CI**	**P**	**HR**	**95% CI**	**P**

Age, years						

≤ 50	1			1		

>50	1.01	0.80-1.29	0.91	1.06	0.82-1.36	0.66

T stage						

T1-T2	1			1		

T3-T4	1.56	1.20-2.04	0.001	1.41	1.05-1.89	0.02

No. of axillary nodes involved						

≤ 3 nodes	1			1		

>3 nodes	1.42	1.12-1.80	0.003	1.43	1.10-1.87	0.007

HR status						

negative	1			1		

positive	0.49	0.39 - 0.62	<0.001	0.62	0.48-0.81	<0.001

HER-2 status						

negative	1			1		

positive	0.80	0.60 - 1.10	0.11	0.69	0.51-0.92	0.01

Adjuvant endocrine therapy						

no	1			1		

yes	0.68	0.54-0.86	0.001	0.94	0.70-1.25	0.66

Adjuvant chemotherapy						

no	1			1		

yes	1.16	0.90-1.50	0.26	1.18	0.90-1.53	0.23

Relapse free interval						

≤ 2 yr	1			1		

>2 yr	0.58	0.46-0.73	<0.001	0.89	0.69-1.15	0.37

Site of first recurrence						

non visceral	1			1		

visceral	1.77	1.40-2.24	<0.001	1.77	1.38-2.27	<0.001

Brain metastatasis at diagnosis						

no	1			1		

yes	1.91	1.09-3.33	0.02	2.33	1.30-4.20	0.005

AIs and/or trastuzumab for recurrent disease						

no	1			1		

yes	0.38	0.29-0.50	<0.001	0.46	0.33-0.63	<0.001

Time period						

cohort A	1			1		

cohort B	0.43	0.34 - 0.55	<0.001	0.70	0.63-0.92	0.01

Abbreviations: HR, hormone receptor; HER-2, human epidermal growth factor receptor 2; AI, aromtase inhibitors

### Influence of HER-2 and HR status on survival between the two cohorts

**Figure 2:**
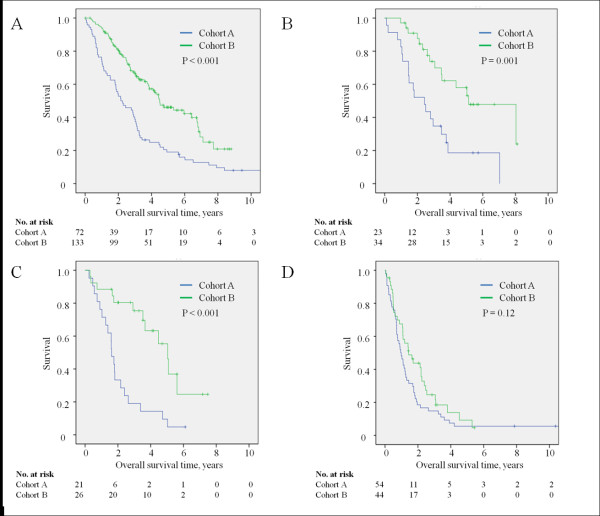
**Kaplan-Meier curves showing the overall survival rates for patients with (A) HR-positive/HER-2-negative, (B) HR-positive/HER-2-positive, (C) HR-negative/HER-2-positive, and (D) HR-negative/HER-2-negative tumors within the two cohorts**.

We also performed a subgroup analysis in order to explore the association between the recent improvement in the survival of recurrent breast cancer patients and the breast cancer subtype as determined by HR and HER-2 status. For this purpose, patients were categorized into four subgroups based on their HR and HER-2 expression patterns. There have been 133 deaths among patients of the HR-positive/HER-2-negative subgroup; 34 deaths in the HR-positive/HER-2-positive subgroup; 32 deaths in the HR-negative/HER-2-positive subgroup; and 87 deaths in the HR-negative/HER-2-negative subgroup. The Kaplan-Meier curves illustrating the overall survival rates for each subgroup within the two cohorts are shown in Figure 2. For the HR-positive/HER-2-negative subgroup, the median survival in cohort A was significantly lower than that in cohort B (2.2 vs. 4.5 years, P < 0.001; Figure 2A; n = 205). For the HR-positive/HER-2-positive subgroup, the survival rate within cohort A was significantly lower than in cohort B (2.4 vs. 5.1 years, P = 0.001; Figure 2B; n = 57). For the HR-negative/HER-2-positive subgroup, the median survival rate within cohort A was significantly lower than that in cohort B (1.6 vs. 5.0 years, P < 0.001; Figure 2C; n = 47). In contrast, for the HR-negative/HER-2-negative subgroup, the prognosis remained poor over time and no significant survival improvement was seen between the two cohorts (1.0 vs. 1.4 years, P = 0.18, Figure 2D; n = 98). To clarify the effect of time period and AI/trastuzumab treatment on survival in each subtype, multivariate analyses in the 4 subgroups were performed (Table 4). When AI/trastuzumab and the time period were included in the same model, AI and/or trastuzumab was a significant favorable prognostic factor in the HR-positive/HER-2-negative subgroup (AIs and/or trastuzumab for recurrent disease: yes vs. no: HR = 0.37, P < 0.001) and the recent time period was significantly associated with prolonged survival in the HR-positive/HER-2-positive (cohort B vs. cohort A: HR = 0.39, P = 0.009) and HR-negative/HER-2-positive subgroups (cohort B vs. cohort A: HR = 0.12, P < 0.001). When AI/trastuzumab and the time period were separately analyzed, each factor was a strong prognostic factor in the HR-positive and/or HER-2-positive subtype but not in the HR-negative and HER-2-negative subtype in the presence of other confounders.

**Table 4:** Cox multivariate analysis of the survival in recurrent breast cancer by breast cancer subgroup

	HR-positive/HER-2-negative		HR-positive/HER-2-positive		HR-negative/HER-2-positive		HR-negative/HER-2-negative	
	**HR (95% CI)**	**P**	**HR (95% CI)**	**P**	**HR (95% CI)**	**P**	**HR (95% CI)**	**P**

Age (>50 vs ≤50)	1.21 (0.85-1.73)	0.19	0.27 (0.11-0.70)	0.006	1.32 (0.47-3.73)	0.60	0.70 (0.44-1.13)	0.14

T stage (T3,4 vs T1,2)	2.00 (1.33-3.02)	0.001	2.74 (1.08-6.92)	0.03	1.40 (0.61-3.20)	0.42	1.22 (0.74-2.00)	0.44

No. of axillary nodes involved (3 < nodes vs ≤3 nodes)	1.18 (0.77-1.79)	0.45	0.93 (0.38-2.29)	0.88	2.42 (0.99-5.89)	0.054	1.52 (0.99-2.34)	0.057

HR status (positive vs negative)	NA	NA	NA	NA	NA	NA	NA	NA

HER-2 status (positive vs negative)	NA	NA	NA	NA	NA	NA	NA	NA

Adjuvant endocrine therapy (yes vs no)	1.01 (0.64-1.59)	0.98	0.72 (0.29-1.77)	0.47	NA	NA	NA	NA

Adjuvant chemotherapy (yes vs no)	1.50 (1.03-2.19)	0.04	1.69 (0.70-4.08)	0.24	1.76 (0.68-4.53)	0.24	0.74 (0.40-1.36)	0.33

Relapse free interval (>2 yr vs ≤ 2 yr)	1.00 (0.67-1.50)	0.98	0.64 (0.30-1.37)	0.25	0.26 (0.11-0.61)	0.002	0.91 (0.55-1.52)	0.73

Site of first recurrence (visceral vs non visceral)	1.67 (1.17-2.39)	0.005	2.95 (1.36-6.41)	0.006	1.81 (0.77-4.27)	0.18	1.77 (1.12-2.79)	0.01

Brain metastatasis at diagnosis (yes vs no)	2.22 (0.68-7.27)	0.19	0.58 (0.06-5.33)	0.63	6.02 (1.87-19.3)	0.003	2.76 (0.97-7.83)	0.056

AIs and/or trastuzumab for recurrent disease (yes vs no)	0.37 (0.25-0.53)	<0.001	0.55 (0.10-2.96)	0.49	0.61 (0.18-2.05)	0.42	NA	NA

Time period (cohort B vs cohort A)	0.81 (0.50-1.30)	0.38	0.39 (0.19-0.79)	0.009	0.12 (0.05-0.29)	<0.001	0.80 (0.50-1.29)	0.36

Abbreviations: HR, hormone receptor; HER-2, human epidermal growth factor receptor 2; AI, aromtase inhibitors

## Discussion

In this retrospective, single-institution study, we found that the survival of patients with recurrent breast cancer was significantly improved after the introduction of AIs and trastuzumab to the therapeutic regimen after 2001. Also, an improved prognosis was seen in patients with recurrent breast cancer that was related to HR and HER-2 expression. Patients with HR-positive and/or HER-2-positive tumors showed improved survival times after 2001; however, this was not the case for patients with HR-negative/HER-2-negative tumors.

There is a consensus of opinion that the survival of patients with recurrent, or metastatic, breast cancer has improved with the introduction of new agents [9,10,23]; however, it is not clear which particular therapy has been responsible for this improvement. Data from the French comprehensive cancer centers shows that increased survival time is associated with HR expression by the tumor [14]. The hypothesis that the development of endocrine therapy has played an important role in increased survival rates is supported by the results of many randomized trials. Third-generation aromatase inhibitors (AI) recently became available for the treatment of breast cancer, and a number of randomized trials in postmenopausal women with advanced breast cancer have shown the superiority of AI over standard endocrine agents such as tamoxifen and megestrol acetate [24-26]. There seems to be partial non-cross resistance between different types of AIs [22,27]. Exemestane (a steroidal AI) was shown to be effective after the failure of other non-steroidal AIs. In our study, a significant prolongation in survival time was seen in patients with HR-positive tumors within cohort B, which consisted of patients diagnosed and treated after the 2001, the year when AIs were first approved in Japan. This suggests that the survival rates of the HR-positive recurrent breast cancer patients improved due to treatment with AIs.

The introduction of HER-2 targeting therapies is also thought to have played an important role in the increased survival times. In HER-2-positive metastatic, or advanced, breast cancer, several clinical trials showed a significant clinical benefit of trastuzumab as a first-line chemotherapy agent [28,29]. There is also some evidence from retrospective studies that metastatic breast cancer patients with HER-2-positve tumors benefit from the use of trastuzumab beyond disease progression [30,31], and a recent randomized trial showed that continuation of trastuzumab-containing treatment was associated with a significant improvement in the overall response rate and time to progression after disease progression in patients with HER-2-positive metastatic breast cancer [21]. In our study, significant improvements in survival times were seen in patients with HER-2-positive tumors in cohort B, suggesting that initial, or continued, treatment with trastuzumab was responsible.

As shown in previous reports [9,32,33], the presence of conventional adverse prognostic factors was shown to be associated with poor prognosis in this study (Table 3). The presence of visceral metastases, brain metastasis, HR status, and T stage and the number of axillary metastases significantly increased risk of death. However, even after taking these prognostic factors into account, the presence of AIs and/or trastuzumab treatment significantly decreased the hazard of death in multivariate analysis, and one could argue that the introduction of AIs and trastuzumab made the great contribution to the survival improvement. The time period was also shown to be a significant prognostic factor in multivariate analysis and recent time period conferred prolonged survival. There were also major differences between the two cohorts in terms of the other treatments received (i.e., the treatments other than AIs and trastuzumab). Other modern cytotoxic agents such as taxanes, capecitabine, and vinorelbine were used more frequently in cohort B. As shown in previous reports, the introduction of these new drugs for breast cancer treatment appears to have improved the survival rates of recurrent breast cancer patients [9,10]. In addition, bisphosphonates have recently become available for the treatment of skeletal metastases, and have been shown to reduce the risk of bone-related events [34,35]. Palliative care has also progressed. These advances in the treatment for recurrent breast cancer could be the reason why the time period was still a significant factor in this analysis.

As an exploratory analysis, we also investigated the association between survival improvement and breast cancer subtypes defined by HR and HER-2 status. Significant survival improvements were recognized in the HR-positive and/or HER-2-positive subtype after introduction of AIs and trastuzumab (Figure 2). With the introduction of these new agents, the breast cancer subtypes became closely related to the survival of patients with recurrent breast cancer. Regarding the HR-negative/HER-2-negative population, our study was underpowered in terms of demonstrating a significant survival benefit in this subtype, and indeed the prognosis was worse for this group than for the other tumor subtypes. This subtype is reported to be associated with aggressive behavior and poor prognosis [18,36]. In a recent retrospective study of HR-negative/HER-2-negative breast cancer patients treated with modern chemotherapy, the median survival time from the diagnosis of metastatic lesions was only 13.3 months [33]. This is far shorter than the median survival times reported in previous studies [9,10,37]. Exploratory analysis of data from the Cancer and Leukemia Group B 9342 study, which tested three doses of paclitaxel in women with advanced disease, showed that HR-negative/HER-2-negative tumors are associated with shorter overall survival rates compared with other subtypes [38]. Our results showed that patients with HR-negative/HER-2-negative tumors had no significant prolongation of survival after trastuzumab treatment; the prognosis of these patients was only 1.4 years, even after the introduction of AI, trastuzumab and other new agents such as taxanes, vinorelbine and capecitabine. However, it is important to mention that this subgroup analysis was not part of the original study design, and that there was not enough statistical power to lead to a definitive conclusion. Therefore, this finding must be interpreted with caution.

We acknowledge that our study has several important limitations. First, this was a retrospective study, and thus subject to all the biases inherent to a retrospective design. Some degree of bias was present in the unadjusted analysis due to the weighting of more favorable prognostic factors, reduced number of lymph node metastases, reduced incidence of visceral metastasis, increased incidence of HR-positive, and longer RFI, in the groups that developed recurrent disease in more recent years. Earlier reports confirmed that patients with metastatic disease who had shorter disease-free survival times, HR-negative tumors, and visceral metastases, had significantly worse survival rates [32]. Although the time period was shown to be a significant prognostic factor in the multivariate analysis, one could argue that these biases led to the survival improvement over time. Second, only 72% of the patients who were treated for recurrent disease were analyzed in this single-institution retrospective study. We cannot exclude the possibility that the analyzed population could have been biased as compared with the original cohort. Third, this study employed only a relatively small sample from a single institute and thus lacked the statistical power to lead to a definitive conclusion. Forth, information of survival curve beyond 4 years shown in Figure 2 may be little value due to the low numbers at risk. Further examination with larger number of cases is warranted to certify our findings.

## Conclusion

In conclusion, our study suggests that the survival rates of women with recurrent breast cancer significantly improved after the introduction of AI and trastuzumab, and that the survival improvement was most pronounced in the HR-positive and/or HER-2-positive subgroup. The prognosis for HR-negative/HER-2-negative recurrent breast cancer patients was still poor and development of new therapies for this population is warranted.

## Abbreviations

AIs: third generation aromatase inhibitors; HR: hormone receptor; TAM: tamoxifen; ER: estrogen receptor; RFI: relapse free interval; ELISA: enzyme immunoassay method; IHC: immunohistochemical analysis; CI: confidence intervals.

## Competing interests

The authors declare that they have no competing interests.

## contributions of the authors

HK planned the data analysis, carried out all statistical analyses, interpreted the results, and drafted the manuscript. YN contributed to the interpretation of the data and revised the manuscript for important intellectual content. SS, CK, KT and SN participated in the design of the study and revised the manuscript. Pathologists KT and KN evaluated the results of the immunohistochemical analysis. SO conceived of the study, participated in its design and coordination, and revised the manuscript for important intellectual content. All authors read and approved the final manuscript.

## Pre-publication history

The pre-publication history for this paper can be accessed here:

http://www.biomedcentral.com/1471-2407/11/118/prepub
